# Occurrence of the Tamarix Leafhopper, *Opsius stactogalus* Fieber (Hemiptera: Cicadellidae), in Argentina

**DOI:** 10.1673/031.010.2301

**Published:** 2010-03-23

**Authors:** Eduardo G. Virla, Guillermo A. Logarzo, Susana L. Paradell

**Affiliations:** ^1^ PROIMI-Biotecnología, División de Control Biológico, Avenida Belgrano y Pasaje Caseros, T4001 MVB, San Miguel de Tucumán, Tucumán, Argentina; ^2^ USDA-ARS, South American Biological Control Laboratory, Bolivar 1559 (1686), Hurlingham, Buenos Aires, Argentina; ^3^ Facultad de Ciencias Naturales y Museo de La Plata, Universidad Nacional de La Plata, Paseo del Bosque sin número, CP 1900, La Plata, Buenos Aires, Argentina

**Keywords:** distribution, *Tamarix*, tamarisk leafhopper, invasive species

## Abstract

The paleartic tamarix leafhopper, *Opsius stactogalus* Fieber (Hemiptera: Cicadellidae), can reduce the growth of tamarisk due to the aggregate feeding imposed by their populations. The species was mentioned for Argentina in Metcalf's catalogue (1967) without locality or region reference, and the contributions on Cicadellidae published by many authors after Metcalf omitted this distributional data. Populations of *O. stactogalus* on *Tamarix* sp. were found in 12 sites between 28° 48′ to 39° 17′ S and 64° 06′ to 70° 04′ W, located in both the Neotropical and Andean biogeographic regions.

## Introduction

Species of *Tamarix* (Caryophyallales: Tamaricaceae) are native of Eurasia and Africa and, due to their extraordinary biological features and adaptive capabilities (Fornasari 2004), have been introduced and established in 44 countries throughout the world.

At least four species of *Tamarix* (*T. gallica*, *T. ramosissima*, *T. chinensis* and *T. parviflora*) and their hybrids occur in Argentina (Gaskin and Schaal 2003; Natale et al. 2008). They were introduced to cover human necessities, mostly as windbreaks, to fix dunes, to control soil erosion, to supply shade, and in some desert places tamarisks are the lone surviving woody plants (León 2006).

However, tamarisk is now an alien plant that invaded the Pampean grasslands, mostly in coastal dunes or in riparian habitats, where it is more invasive, spreading over natural or semi-natural ecosystems (Zalba and Villamil 2002). In addition, tamarisk became a noxious weed in Llancanelo and Guanacache lagoons, both protected RAMSAR sites in Cuyo region, because its invasiveness produces soil salinization, water obstructions, and habitat modifications altering the riparian/wetland trophic structure (Sosa 2003). Consequences of tamarisk's invasiveness, such as water uptake and low diversity of dependent wildlife, have made it the target of classical biological control in the United States (Fornasari 1997, 2004).

Few insects are cited as affecting *Tamarix* spp. in Argentina: *Ceroplastes* sp., *C. formicarius* Hempel and *Coccus hesperidum* (L.) (Hem: Coccidae), *Automeris aspersa* (Felder) (Lep: Saturniidae), *Oiketicus platensis* Berg (Lep: Psychidae), *Bostrichopsis uncinata* Germ. (Col: Bostrichidae), and *Praxithea deroudei* (Chabrillac) (Col: Cerambicidae)(Cordo et al. 2004).

## Methods and Materials

The geographic range of wild populations of *Tamarix* spp. cover most of Argentina, except the northeast (Natale et al. 2008). Seven out of 12 provinces where *Tamarix* spp occurs were surveyed. All the specimens were collected using entomological nets and manual aspirators. Specific identification was made using the keys provided by Oman (1936) and Linnavuori (1959), using both external and male genitalia's characters. Voucher specimens of *O. stactogalus* resulting from this study are deposited in the collections of the Museo de Ciencias Naturales de La Plata, Buenos Aires (MLPA) and Fundación e Instituto Miguel Lillo at San Miguel de Tucumán (IMLA), Argentina.

## Results

*Gonatocerus tuberculifemur* Ogloblin (Hym: Mymaridae) is an egg parasitoid candidate for the control of *Homalodisca vitripennis* (Hem: Cicadellidae) in the United States (Jones et al. 2005). During the process of selecting leafhopper species for the study of their host range, abundant populations of a small leafhopper were found on *Tamarix* sp. in several sites in the Pampas and Monte provinces (Neotropical region, Chacoan subregion), and central and subandean patagonia provinces (Andean region, patagonian subregion) (*sensu* Morrone 2001); it was identified as the Holartic species *Opsius stactogalus* Fieber, 1866 (Hemiptera: Cicadellidae) known as the *“Tamarix* leafhopper”.

*Opsius stactogalus* (Deltocephalinae: Opsiini) is a leafhopper native to Europe and is strongly associated with the shrubby tree tamarisk (Wiesenborn 2001, 2002). The distribution is primarily Paleartic, but now nearly cosmopolitan, and always associated with tamarisk. Linnavouri & De Long (1977) found this species associated with *Casuarina* sp. in Chile. This species was previously mentioned by Metcalf (1967) for “Argentina” without locality or region reference, but the known distribution that was published by several authors subsequent to the Metcalf catalogue (Linnavuori 1959; Linnavouri and De Long 1977; Evans 1977; Oman et al. 1990; Zanol 2006) did not mention Argentina. The *Tamarix* leafhopper is a sap-feeder, and the aggregate feeding imposed by their populations can reduce tamarisk's growth (Liesner 1971, as cited by Wiesenborn 2002). Given the plant's pervasiveness and its undesirable qualities, new distributional data were obtained.

### Examined material

ARGENTINA: Rio Colorado (Rio Negro province) (38° 59′ 45.1 S 64° 06′ 07.6 W, elevation: 91 MASL), 21.II.07, 3 females, 1 nymph, Virla & Logarzo cols. (MLPA); Choele Choel (Rio Negro prov.) (39° 17′ 22.6 S 65° 40′ 07.4 W, 126 MASL), 4 females, 1 male, Virla & Logarzo cols. (MLPA); Chimpay (Rio Negro prov.) (39° 10′ 08.7 S 66° 08′ 48.8 W, 154 MASL), 22.II.07, 3 females, 2 males, Virla & Logarzo cols. (MLPA); Neuquén city (Neuquén prov.) (38° 57′ 18.2 S 68° 08′ 42.9 W, 274 MASL), 22.II.07, 5 males, 3 females, 2 nymphs, Virla & Logarzo cols. (MLPA); Zapala (Neuquén prov.) (38° 59′ 4 S 70° 04′ 5 W, 1020 MASL), 10.II.08, 2 females, 4 males, 2 nymphs, Virla col. (IMLA); Villa E1 Chocón (Neuquén prov.) (39° 15′ 43.4 S 68° 46′ 39.1 W, 412 MASL), 02.II.08, 3 females, 3 nymphs, Virla col. (IMLA); Santa Isabel (La Pampa prov.) (36° 22′ 27.2 S 67° 04′ 06.8 W, 303 MASL), 18.II.08, 3 males, 10 females, Logarzo col. (IMLA); Algarrobo del Aguila (La Pampa prov.) (36° 24′ 23.9 S 67° 08′ 22.3 W, 301 MASL), 10.II.08, 2 males, 1 female, 1 nymph, Virla col. (IMLA); San Rafael (Mendoza prov.) (34° 45′ 51 S 68° 24′ 41.4 W, 674 MASL), 11–12.II.08, 8 males, 12 females, 14 nymphs, Virla col. (IMLA); Aimogasta (La Rioja prov.) (28° 34′ 18 S 66° 48′ 03 W, 847 MASL), 24.XII.07, 3 females, 3 males, 2 nymphs (IMLA), Virla col.; Villa Mazán (La Rioja) (28° 39′ 57.9 S 66° 31′ 24.2 W, 652 MASL), 10.II.08, 24 females, 65 males, 46 nymphs, Virla col. (IMLA); Chumbicha (Catamarca prov.) (28° 48′ 56 S 66° 14′ 50 W, 506 MASL), 21.XII.07, 4 females, 2 males, 3 nymphs, Virla col. (IMLA).

## Discussion

*Opsius stactogalus* was found in all provinces sampled, except Córdoba (National Road n°7 km 526, near Gral. Levalle), in 12 out of the 13 sites surveyed. In all sites *O. stactogalus* was very abundant, however damage produced by this leafhopper on the sampled plants was not observed. Therefore, the potential usefulness of this hopper as a biocontrol agent for the invasive tamarisk populations established in Argentina is doubtful. *Opsius stactogalus* was also recorded in the United States of America, and again it did not cause significant damage to *Tamarix* spp. (De Loach 2001).

These findings encourage exploration for possible natural enemies of *O. stactogalus*, either natives of Argentina or others that arrived with this leafhopper, in order to better understand the community history of tamarisk in Argentina.

## Abbreviations

IMLA: Fundación e Institute Miguel Lillo at San Miguel de Tucumán, Argentina;
MLPA: Museo de Ciencias Naturales de La Plata, Buenos Aires;
MASL: meters above sea level
